# Keynote 2: How our social world shapes physical activity and exercise

**DOI:** 10.1093/eurpub/ckad133.003

**Published:** 2023-09-11

**Authors:** Tegan Cruwys

**Affiliations:** College of Health and Medicine, Australian National University

## Abstract

While research on the determinants of physical activity has ostensibly used draws on a biopsychosocial model, in reality, the social determinants of physical activity have been relatively neglected. In my talk, I will provide an overview of the empirical evidence for how social phenomena - including our group memberships, social norms, and leadership - shape peoples' participation in, exertion during, and enjoyment of, physical activity. As well as reviewing the evidence for these effects, my talk will also explore how and why: what are some potential underpinning mechanisms for these robust links? The theoretical and practical implications of these findings will be discussed, with a focus on how social psychology can be harnessed for health behaviour interventions.

